# Linc2function: A Comprehensive Pipeline and Webserver for Long Non-Coding RNA (lncRNA) Identification and Functional Predictions Using Deep Learning Approaches

**DOI:** 10.3390/epigenomes7030022

**Published:** 2023-09-15

**Authors:** Yashpal Ramakrishnaiah, Adam P. Morris, Jasbir Dhaliwal, Melcy Philip, Levin Kuhlmann, Sonika Tyagi

**Affiliations:** 1 Central Clinical School, Monash University, Melbourne, VIC 3000, Australia; 2 School of Computing Technologies, Royal Melbourne Institute of Technology University, Melbourne, VIC 3000, Australia; 3 Monash Data Futures Institute, Monash University, Clayton, VIC 3800, Australia; 4 Faculty of Information Technology, Monash University, Clayton, VIC 3800, Australia

**Keywords:** lncRNA, non-coding RNA, machine learning, functional annotation, deep learning

## Abstract

Long non-coding RNAs (lncRNAs), comprising a significant portion of the human transcriptome, serve as vital regulators of cellular processes and potential disease biomarkers. However, the function of most lncRNAs remains unknown, and furthermore, existing approaches have focused on gene-level investigation. Our work emphasizes the importance of transcript-level annotation to uncover the roles of specific transcript isoforms. We propose that understanding the mechanisms of lncRNA in pathological processes requires solving their structural motifs and interactomes. A complete lncRNA annotation first involves discriminating them from their coding counterparts and then predicting their functional motifs and target bio-molecules. Current in silico methods mainly perform primary-sequence-based discrimination using a reference model, limiting their comprehensiveness and generalizability. We demonstrate that integrating secondary structure and interactome information, in addition to using transcript sequence, enables a comprehensive functional annotation. Annotating lncRNA for newly sequenced species is challenging due to inconsistencies in functional annotations, specialized computational techniques, limited accessibility to source code, and the shortcomings of reference-based methods for cross-species predictions. To address these challenges, we developed a pipeline for identifying and annotating transcript sequences at the isoform level. We demonstrate the effectiveness of the pipeline by comprehensively annotating the lncRNA associated with two specific disease groups. The source code of our pipeline is available under the MIT licensefor local use by researchers to make new predictions using the pre-trained models or to re-train models on new sequence datasets. Non-technical users can access the pipeline through a web server setup.

## 1. Introduction

LncRNAs, which account for approximately 80% of RNA transcribed in cells, are known to play diverse and crucial functions, making them emerge as master regulators of gene expression [1,2]. These functions are carried out through various interaction mechanisms with other bio-molecules at the transcription, translation, or epigenetic level [3]. However, it is important to note that not all lncRNAs possess dedicated functions. Some of them may be biological and technical artifacts or simply transcribed introns and isoforms of protein-coding genes lacking any specific regulatory role [4]. Despite the growing discoveries, about 95% of lncRNAs still lack functional annotation [5]. Existing annotation methods and databases show limited consensus [6]. Inconsistencies between databases are a major concern and require newer methods to annotate these RNAs reliably. Moreover, some important genes were excluded in recent versions of databases, despite their association with various diseases [7,8]. Additionally, lncRNA annotations are severely lacking in newly sequenced genomes, necessitating reference-free or ab initio methods for further exploration.

LncRNAs play a significant role in regulating gene expression across various biological processes and pathways, implicating them in numerous diseases. Recent studies highlight how misregulation or mutation in lncRNAs contributes to various diseases [9,10]. For instance, the LncBook database reports that around 80% of experimentally validated lncRNAs [11] are linked to more than 400 diseases, and the LncRNADisease 2.0 database predicts more than 200,000 disease associations [7,8]. Another study that aimed to explore gene–trait linkage suggested that the majority of disease-associated lncRNAs operate independently without the presence of neighbouring genes [12]. Therefore, understanding the functional mechanisms of lncRNA can have far-reaching implications, enabling the early diagnosis, prognosis, prevention, and treatment of several disorders.

### 1.1. Structure to Function

It is popularly known in the protein world that “structure is function”, and the same principle applies to lncRNAs. These molecules exhibit functional mechanisms akin to proteins as they can fold into a secondary (2D) and tertiary (3D) structure containing multiple structural domains. They function by structurally binding to their target interactome, forming complexes [13,14,15]. These interactions enable lncRNA to epigenetically regulate cellular biology, forming a layer of genomic programming on top of the coding genes. The target interactome comprises bio-molecules such as DNA, RNA, proteins, and other ligands and is mainly influenced by their 2D structure [16].

Consequently, the secondary structures are subject to evolutionary constraints, resulting in higher conservation than their corresponding primary sequences [17]. Although structural conservation in lncRNAs has always been a matter of conflict, there is still no strong evidence to prove the lack of conservation in lncRNAs. A study utilizing covariance pattern analysis emphasized the necessity for stronger evidence regarding conservation in secondary structures, utilizing a selection of well-studied functional lncRNAs [18]. However, another study demonstrated the conservation in lncRNA structures by employing improved parameters in R-scape [19]. Additionally, the implementation of the CaCoFold (Cascade variation/co-variation Constrained Folding) algorithm’s predictions is promising, as they show consistency with the modelled structures from crystallography [20]. These findings further suggest that the lncRNA follows weaker conservation patterns than protein-coding transcripts, necessitating the use of more specialized computational algorithms for their study.

### 1.2. Interactome

Thus, it is now clear that determining lncRNA folding and its structural domains are crucial in unlocking its function in disease aetiology and determining its interactome [16,21]. Some of the known lncRNA–DNA interactions involve forming structures such as R-loops [22] and triple helices [23] that, in turn, are involved in transcriptional and post-transcriptional regulation, chromatin remodelling, and DNA repair [23]. Thus, a lncRNA transcript with high triplex-forming potential (TFP) is more likely to be involved in these mechanisms either in cis or in trans orientation to the target transcript.

RNA-Binding Proteins (RBPs) form RNA–protein complexes by binding to lncRNAs, and these complexes are associated with regulating various cellular pathways [24]. The functions of RBPs include transcription and translation regulation, DNA repair, splicing, apoptosis, and mediating stress responses [25]. By knowing the potential RBPs’ interactions with an lncRNA, it should be possible to predict the functional pathways in which the lncRNA is involved, and these RBP interactions can be estimated via the presence of sequence or structural motifs on it. We have recently reviewed methods for lncRNA–protein interactions, suggesting the computational and machine learning approaches taking the lead [26].

LncRNA interacts with other ncRNA and mRNA to perform their function [27]. LncRNA–miRNA interaction is a well-studied phenomenon resulting in lncRNAs directly regulating the expression levels of a particular gene. MiRNAs can down-regulate the expression of their target mRNAs by binding in their UTR region [28]. LncRNAs can act as competing endogenous RNA (ceRNA), where they compete for miRNA binding along with mRNAs, consequently acting as regulators of corresponding mRNA targets [29,30]. Thus, knowing the possible miRNA interactions of an lncRNA will enable us to know the genes that the lncRNAs can possibly modulate.

LncRNAs also interact with the low-molecular-weight compounds that bind to the structural loci on these transcripts. This provides the opportunity to regulate cellular activity using these compounds. However, the knowledge about these interactions is currently limited. Therefore, in this study, we have not covered these interactions.

We divide the lncRNA annotation problem into two parts: (1) identification, i.e., distinguishing an lncRNA transcript from other non-coding and coding transcripts and (2) annotation, i.e., identifying its structural motifs along with its interacting bio-molecules. We will refer to the set of interacting bio-molecules as the lncRNA “interactome”.

### 1.3. Computational Identification and Annotation of lncRNA

Primary data to identify lncRNA come from high-throughput sequencing (HTS) experiments [31]. The assembled transcripts from an RNA sequencing experiment are further analysed to annotate them as various types of RNA transcripts. It is challenging to obtain transcript features that can be used to distinguish an lncRNA from other RNA transcripts due to their high diversity and lack of comprehensive high-confidence reference data. However, a machine learning model can help identify such generic features in an ab initio manner. When trained existing known lncRNAs examples, it can pick up similar patterns on screening the transcripts obtained in RNA-seq experiments en masse [3,32].

Experimental methods to identify the RNA interactome are expensive and time-consuming and cannot be performed for each cell type or state. Moreover, the expression level of these lncRNAs is very low compared to mRNAs and also tissue-specific in nature, which poses further challenges for in vitro methods. Hence, in silico approaches are a favourable choice to predict the lncRNA interactome.

We recently reviewed existing in silico methods and current challenges in lncRNA identification [6,26]. These identification methods fall into two categories: (a) rule-based machine learning, which requires known features, which can be species-specific or generic, to distinguish lncRNA from other transcripts, and (b) deep learning approaches that do not require pre-coded features and can learn them directly from the sequences. However, access to source code for the existing tools is often restricted posing challenges to retrain on newer datasets for performing cross-species predictions. Additionally, current deep learning techniques lack interpretability, preventing the examination of the transcript features that influenced the predictions. Furthermore, the current best-performing lncRNA analysis tools do not consider the identification of structural domains and interactomes, which are crucial for complete functional annotation (refer to Table 1).

Like mRNA, a single lncRNA gene can have several transcript isoforms with varying length and sequence content [33,34]. Thus, these transcript isoforms could have different functions due to their target interactome-binding differences.

In this study, we attempt to close the gaps discussed above: (1) the lack of isoform-level analysis; (2) the lack of specialized computational techniques for comprehensive functional annotation; (3) the limitations of reference-based methods for cross-species predictions; and (4) the lack of accessibility to the complete source codes for re-training on newer datasets. In this paper, we present a comprehensive pipeline implemented as a web server to functionally annotate disease-associated lncRNA at the isoform level. This software is also available as a standalone application for local use where researchers can evaluate distinct sets of features of lncRNA of interest.

## 2. Methods

We have implemented two types of machine learning models in our pipeline that will be discussed in detail in the following section.

### 2.1. Rule-Based Machine Learning Approach (ANN Models)

A rule-based approach allows us to evaluate the effect and importance of pre-computed features in classifying sequences as lncRNA or not.

#### 2.1.1. Data

Open-source repositories store lncRNA data with diverse levels of detail. Some focus on identified lncRNAs and sequences, while others include structural, functional, and disease association information. Data are collected through manual curation, low/high-throughput experiments, in silico predictions, or a mix of these methods. Gathering can be managed through domain experts, crowdsourcing, or computer automation. Due to their diverse origins, direct comparisons between these repositories are complicated [6]. To achieve consensus data from all four repositories, we chose to use the genomic coordinates matching with a tolerance of plus or minus five nucleotide positions. These consensus data, as depicted in Figure S4, yielded 10,179 lncRNA IDs, out of which we could obtain sequences for 9270 non-coding transcripts from GENCODE (Frankish et al. 2019) human release v33 (GRCh38.p13), which was the latest version at that time. Likewise, we selected an equal number of coding transcripts (mRNAs) from human release v34 (GRCh38.p13).

Further, in this direction, we looked for ultra-conserved regions (UCRs) around 65 lncRNA genes implicated in endometriosis, as sourced from the FANTOM-CAT dataset [35]. We did not find any overlap of UCR regions, with the exonic loci of these genes indicating a poor sequence conservation. On the other hand, we observed that seven genes had UCR regions in non-exonic regions. More details are available here: https://gitlab.com/tyagilab/linc2function/-/blob/master/HumanDiseaseUcr/human_disease_ucr.md (accessed on 13 September 2023). UCR may form a part of the lncRNA and, thus, contribute to its secondary structure [16].

#### 2.1.2. Feature Extraction

**Figure 1:**
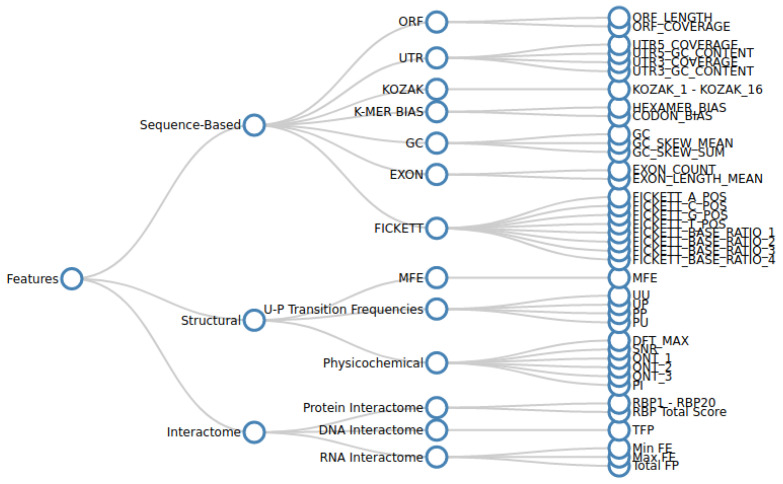
The table caption illustrates a hierarchical tree diagram depicting the organization of various features and their respective categories considered in the study. The initial categorization involves three broad groups: sequence-based, structure-based, and interactome-based features. These groups are further subdivided into more detailed categories in the subsequent level, followed by the enumeration of specific features.

Various sequence-based, structural, and interactome features are extracted from the curated nucleotide sequence data as described in Section 2.1.1. We analysed the effectiveness of each feature in predicting lncRNA forming potential in a standalone manner, as well as in conjunction with other features. For this purpose, we collected as many features as practically possible. A hierarchical tree diagram of the collected features is given in Figure 1.

Sequence-based features quantify the various nucleotide distribution patterns of a transcript. Structural features capture secondary structure characteristics, such as minimum free energy (MFE), paired–unpaired transition frequencies, and physicochemical measures. Interactome features are the measure of the nature and magnitude of the lncRNA interactions with their target RNA, DNA, or protein bio-molecules.

#### 2.1.3. Model Training and Performance Evaluation

The ANN model was built using the python library Keras (v 2.12.0) with TensorFlow (v 2.12.0) library in the backend. A balanced dataset is generated by randomly selecting an equal number of coding transcript sequences from the same source. Combining the coding and non-coding sequences and accounting for missing transcripts in the GENCODE file resulted in 18,540 records. Training data constitute 80% of the records, and the remaining 20% of records are used for validation. As described in Table S1, we have built four different types of models, namely, human-specific basic with the top ten features (HSB), human-specific standard with all 35 features (HSS), specific-agnostic basic with the top ten species-agnostic features (SAB), and specific-agnostic standard with all 33 species-agnostic features (SAS). The top features are selected using a method called Recursive Feature Elimination (RFE) shown in Supplementary Figure S3.

All of the four models are used for prediction, where the classes (positive and negative) of the validation data set are predicted and compared against the true labels. We calculated Precision, Recall, F1-Score, and ROC-AUROC metrics to evaluate the performance of the model.

### 2.2. Deep Learning-Based Approach (LRN2 Model)

Deep learning models utilize one-hot encoded sequences as input, eliminating the need for manually obtained features. By doing so, these models can autonomously derive crucial attributes to distinguish classes ab initio from the supplied sequences. Moreover, this approach’s independence from a restrictive predefined set of features potentially enhances its effectiveness in differentiating the classes.

#### 2.2.1. Modification of Existing Model and Recoding

After conducting an extensive literature survey, we decided to adopt lncRNAnet, a deep learning approach, as an alternative to our previous rule-based method for lncRNA identification. Although lncRNAnet showed promising results in previous studies, our efforts to obtain the complete source code from the authors were unsuccessful. Regrettably, we were only able to access their pre-trained lncRNAnet model for testing purposes. This limitation prevented us from conducting evaluations on sequences from different species or with varying genome release versions.

Thus, we developed the above model based on their four-phase algorithm described in [36]: bucketing, ORF indicator detection, sequence encoding, and lncRNA learning. Transcript sequences are grouped into buckets based on their lengths, determined using the bucket width parameter. We improved the algorithm by selecting buckets to ensure all sequence lengths are present throughout each epoch’s training, in contrast to the original method’s random selection. This change prevents bias towards certain sequence lengths, as previously observed when smaller buckets were depleted early in an epoch, leaving only larger ones for later training stages. Then, for each sequence, the ORF indicator is identified before pre-padding the transcript sequence and its ORF indicator to match the maximum sequence length of each bucket. Finally, the lncRNAs are learned when the neural network trains the sequence data. Henceforth, we call this model *LRN2* (lncRNAnet2).

#### 2.2.2. Model and Training and Performance Evaluation

To match the model testing conditions used by the original authors, we selected 24,500 lncRNA and mRNA transcripts between 200 and 3000 nucleotides each from the human release version of GENCODE human release v25 (GRCh38.p7) to train and evaluate the model’s performance. However, to overcome the high training time due to variable sequence lengths, the model uses a bucketing technique that sorts each sequence based on its lengths. Thus, the bucket width is an important parameter. In this paper, we set the width to 500, as suggested by the authors. For model development, we split the training and testing datasets into 80% and 20% portions.

### 2.3. Benchmarking and Comparative Analysis of Models

#### 2.3.1. Comparison of ANN with Other Rule-Based Approaches

A comparative analysis was conducted on three machine learning techniques: Artificial Neural Network (ANN), Support Vector Machines (SVMs), and Naive Bayes (NB). Each model was built for classifying lncRNA using 10-fold cross-validation, utilizing the entire dataset with the corresponding features. The resulting accuracies of the three modelling techniques are visualized as a box plot.

#### 2.3.2. lncRNA Identification Using Different Models

Both the ANN and Deep Learning models were evaluated using a set of 4000 randomly sampled mRNA and lncRNA transcripts each.

#### 2.3.3. Cross-Species Comparison

To perform cross-species lncRNA predictions using the ANN model, we used two groups of models: human-specific and species-agnostic. The first group comprised the HSB and HSS models, while the second group comprised the SAB and SAS models. To test the cross-species predictions of our models, we shortlisted seven species, namely mouse, zebrafish, fruit fly, roundworm, yeast, wheat, and seavase transcripts covering a wide evolutionary spectrum. The mouse data were obtained from the GENCODE [37] database and all the other species data were taken from the Ensembl [38] repository.

### 2.4. lncRNA Annotation

This step involves predicting the secondary structure of the identified lncRNA sequences followed by interactome predictions.

We enhanced an existing Deep Neural Network called SPOT-RNA [39] to predict two-dimensional structures. Our adaptation of the SPOT-RNA addresses the constraints of the original version, which was restricted to sequences of 500 nucleotides or fewer due to computational constraints [39]. To accommodate longer sequences, we employ a strategy where the utility generates multiple predictions. This is achieved by segmenting the input into overlapping sequences of manageable lengths, followed by the integration of individual predictions. Illustrated in Figure S5, this sliding-window methodology facilitates real-time predictions.

We now describe how we obtained the interactome for the sequence provided. LncRNA–DNA interactions were measured as the triplex-forming potential (TFP). This was obtained using TriplexFPP [40], a deep learning-based utility. Similarly, for lncRNA–protein pairs, we used RBPDB [41] to scan sequences for RBP-binding sites. RIblast [42] was used to determine these RNA–RNA interactions. The function of an lncRNA is determined by its structure, and its sequence plays very little role.

The identification and annotation modules were put together as a pipeline called *linc2function*.

## 3. Results and Discussion

### 3.1. ANN Outperforms Other Rule-Based Models

**Figure 2:**
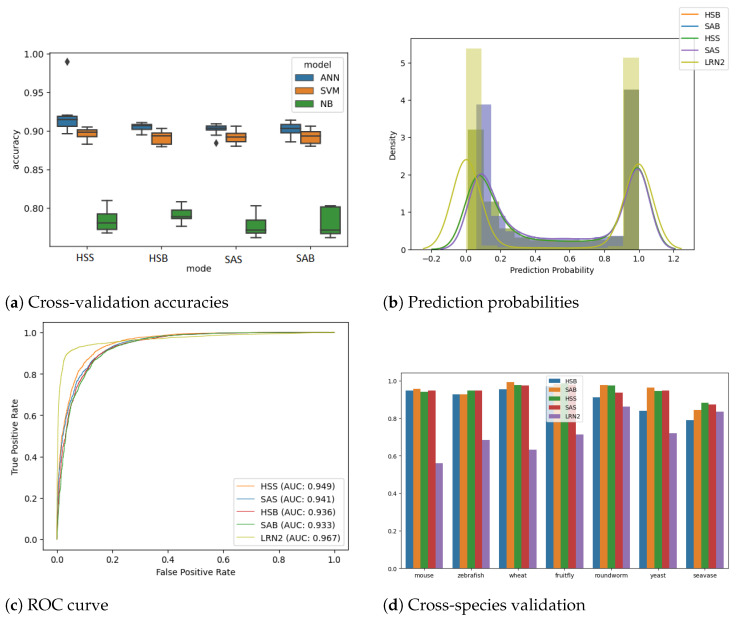
(**a**) This panel presents box plots comparing the 10-fold cross-validation accuracies of the Artificial Neural Network (ANN) against other rule-based machine learning techniques, namely Support Vector Machine (SVM) and Naive Bayes (NB), across four feature sets: HSB, HSS, SAB, and SAS. (**b**) The histogram displayed here illustrates the distribution of prediction probabilities on test data generated using the linc2function models, including HSB, HSS, SAB, SAS, and LRN2, all of which were applied to randomly selected human transcripts sourced from the Gencode database. Transcripts with predictions closer to 1.0 are labelled as non-coding, while those closer to 0.0 are labelled as coding transcripts. (**c**) The ROC curve depicted in this sub-plot represents the testing performance of linc2function models evaluated on randomly selected human transcripts from Gencode. The Area Under the Curve (AUC) values for each model are as follows: HSB model—0.936, HSS model—0.949, SAB model—0.933, SAS model—0.941, and LRN2 model—0.967. Overall, the models demonstrate AUC values above 0.93 on human transcripts, with LRN2 outperforming the others. (**d**) The cross-species analysis of the prediction performance, measured using the AUROC values, of the linc2function models is assessed across eight different species: fruit fly, human, mouse, roundworm, seavase, wheat, yeast, and zebrafish (in no particular order). A more detailed version of this chart is provided in the supplementary section in Figure S7.

Initially, the ANN models were also compared with conventional machine learning models, namely the SVM classifier, a type of generative model, and the NB classifier, which is an example of a discriminative model that performs k-fold cross-validation (k = 10) on all four feature sets, and the results are plotted in Figure 2a. The ANN model performed better than the SVM and NB classifiers in all the modes; hence, we decided to use ANN thereafter.

### 3.2. ANNand RNN Models

As anticipated, when validated on unseen human transcripts, the human-specific model performs marginally better than the corresponding species-agnostic model. The decision to relinquish a small fraction of performance is taken with the aim of generalizing the model for cross-species predictions. The prediction probability distribution (Figure 2b) shows a good separation between the classes. It can also be observed that the models perform identically in predicting coding transcripts. However, the performance for noncoding transcripts varies slightly, with HSS giving the best accuracy, followed by SAS, HSB, and SAB. We evaluated HSS, HSB, SAS and SAB ANN models using k-fold cross-validation (k = 10) to achieve accuracies [mean (+/− std dev)] of 91.13% (+/− 0.54%), 90.47% (+/− 0.63%), 90.21% (+/− 0.77%) and 90.47% (+/− 0.58%), respectively. The testing ROC curve is drawn for all three models (see Figure 2c). Further, the performance evaluation of all the models is summarized in Table 2, which presents key metrics, including precision, recall (also known as sensitivity), specificity, F1 score, and AUROC. These metrics collectively provide an informative assessment of the models’ effectiveness [43,44].

### 3.3. Species Agnostic Models Perform Better at Cross-Species Analysis

It is observed that both the models are able to consistently achieve AUC of over 0.94 over different species, as shown in Figure S7. As expected, the HS model performed well on mammalian transcripts, i.e., human and mouse, but on all the other species, including vertebrates such as zebrafish, the SA model’s prediction performance was superior. This demonstrates the model’s capability of performing well on other divergent species by capturing the generic characteristics of lncRNA transcripts across species.

### 3.4. Our Pipeline Is Accessible via a User-Friendly Webserver Interface

Here, we combine the lncRNA identification and annotation steps to build the first end-to-end pipeline, called *linc2function*. The pipeline implements a rule-based ANN approach and a deep learning approach adopted from a previously published algorithm [36]. The pipeline can identify the lncRNA-forming potential of a given transcript in a species-agnostic manner, specifically by using non-reference features extracted from its sequence, structure, and interactome. The pipeline can take a FASTA sequence as input, and results are displayed as the HTML output. The first section of the *linc2function* results provides basic details of the predicted transcript, such as the name and length of the input FASTA sequence. Additionally, it contains the lncRNA prediction confidence percentage as predicted from the model selected by the user, along with its potential to form triplexes. The following section contains the SHapley Additive exPlanations (SHAP) values showing importance of the individual features for the ANN models. Next, a comprehensive interactome analysis, encompassing proteins, DNA, and RNA, is carried out and made accessible on a user-friendly web page. Users can effortlessly browse and download the results to suit their needs. Since the web-based utility has restrictions on the sequence length it can process, we offer the source code for the pipeline as an alternative. By installing and utilizing this utility, users can conduct more complex analyses beyond these limitations.

### 3.5. Application of linc2function Pipeline for Comprehensive Disease Associated lncRNA Annotations

In this section, we describe two case studies to annotate the disease association of lncRNAs using *linc2function* pipeline. The public lncRNA-disease association databases were first accessed to obtain sets of lncRNA implicated in diseases as they vary in their reported lncRNA–disease association features [6]. Therefore, we selected a consensus set of lncRNAs supported by three different databases: LncBook, EVLncRNAs, and FANTOM-CAT [26]. The LncBook contains experimentally validated data such as multi-omics data integration, functional annotation, and disease association [11], while the EVLncRNAs provide low-throughput experimental data on functional lncRNAs [45]. On the other hand, the FANTOM-CAT contains the association between single-nucleotide polymorphisms (SNPs) and diseases via a genome-wide association study (GWAS), together with conservation data, to predict the lncRNA-associated diseases [35].

We used two lncRNA disease-associated gene sets for interactome prediction. We chose datasets based on the features we wanted to study. But the pipeline is generic to apply to any disease group.

#### 3.5.1. Case Study 1: Isoform-Level Annotation Reveals Functional Diveristy of Transcript Variants

The first set includes 15 lncRNAs known as key regulators in hereditary haemorrhagic telangiectasia (HHT) disease for demonstrating the isoform-level analysis of lncRNA. We collected all the transcripts for the HHT dataset from the Ensembl database. Eleven out of fifteen lncRNAs had more than one transcript isoforms. Splicing analysis revealed that six transcripts have non-canonical splicing sites on them, and four isoforms were not produced from alternative splicing.

**Figure 3:**
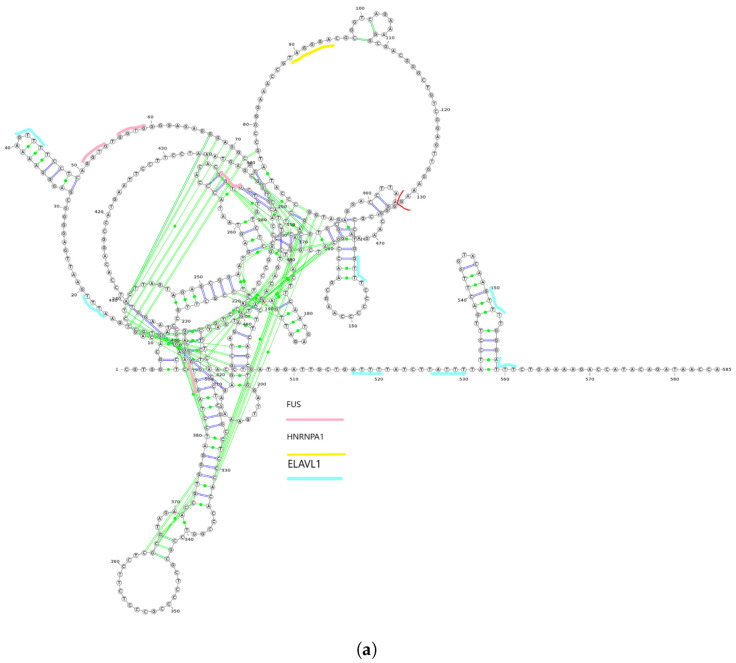

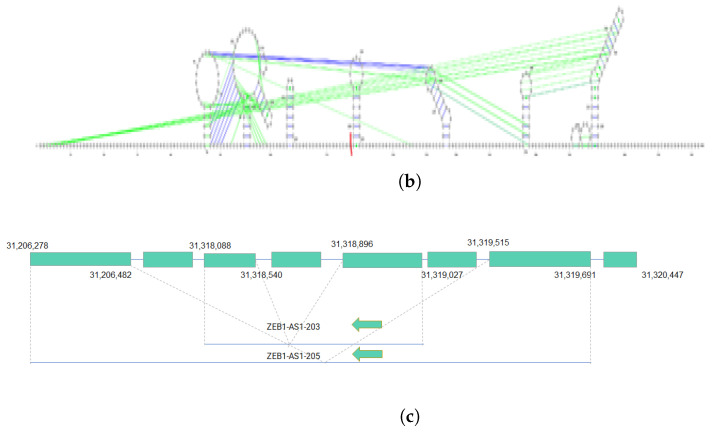
Two isoforms of the ZEB1-AS1 gene with their secondary structures and alternative splicing details. (**a**) The secondary structure of the ZEB1-AS1-203 transcript with protein interactome-binding sites and splicing sites. The red line cutting the loop on the top right near the 130th nucleotide is the splicing site. The pink lines stand for FUS protein-binding sites, the yellow lines stand for HNRNPA1 protein-binding sites, and the light blue lines represent the ELAVL1-binding sites. In the figure, the first exon ranging from nucleotide 1 to 132 has FUS, HNRNPA1 and ELAVL1 protein-binding sites on them. However, the second exon ranging from nucleotide 133 to 585 does not have HNRNPA1 protein-binding site on it; (**b**) The secondary structure of ZEB1-AS1-205 with splicing sites. The red line is the splicing sites. There is no protein interactome predicted by linc2function shared with the two databases with experimental data. (**c**) Alternative splicing of the ZEB1-AS1 gene, showing transcript coordinates and the direction of the two isoforms ZEB1-AS1-203 and ZEB1-AS1-205.

We utilized *linc2function*’s annotation interface (see Figure S6) to generate the annotations for the identified lncRNAs. The secondary structures of ZEB1-AS1-203 and ZEB1-AS1-205 transcripts from the ZEB1-AS1 gene, along with their protein interactome-binding sites and splicing sites, are demonstrated in Figure 3a,b. By combining the protein interactomes and splicing site information (marked in red) based on the structures predicted using *linc2function* (see Figure 3c), we observed that different exons have distinct protein-binding sites, and transcripts from the same gene exhibit variation in protein-binding sites. Additionally, as indicated by *linc2function* (Table S2), the ZEB1-AS1-201 transcript shows coding potential, while ZEB1-AS1-205 does not. This reveals that alternative splicing can lead to different transcripts from the same gene with varied functions.

##### Summary

This case study highlighted secondary structures, i.e., protein interactome-binding sites, for two isoforms of the ZEB1-AS1 gene. Integrating protein interactome and splicing site data revealed specific binding sites in distinct exons and variations in binding sites among transcript isoforms from the same gene. Additionally, the analysis also revealed the coding potential for one of the isoforms. Therefore, our work is the first to demonstrate the functional diversity of lncRNA isoforms due to alternative splicing. This necessitates a genome-scale isoform-level annotation of these transcripts.

#### 3.5.2. Case Study 2: Predicting Interactome Associated with Cancer

The second set consists of 17 lncRNA genes that were reported to be associated with endometriosis cancer. These were annotated using *linc2function*.

Depending on the purpose of the analysis, the *linc2function* annotation generates the following metrics for each considered transcript: Score, Relative Score, RBP Name, Start and End of the interacting region in lncRNA, and the Matching sequence. In our analysis, the RBP names and the number of unique RBPs for each transcript are listed (see Table S3).

The lncRNA interactomes predicted with *linc2function* were cross-checked using different data repositories, namely RBPTD, DECIPHER (DatabasE of genomiC varIation and Phenotype in Humans using Ensembl Resources), OMIM (Online Mendelian Inheritance in Man), and The Human Protein Atlas. RBPTD is a specialized database that identifies cancer-related RBPs in humans, along with a detailed investigation of their functions and abnormalities. On the other hand, DECIPHER is a web-based database that integrates different tools and retrieves information from various bioinformatics resources significant to the variants found in the patients. Finally, OMIM is a free, extensive database for human genes and genetic phenotypes that focuses on traits and disorders.

##### RNA Interactome Validation

**Figure 4:**
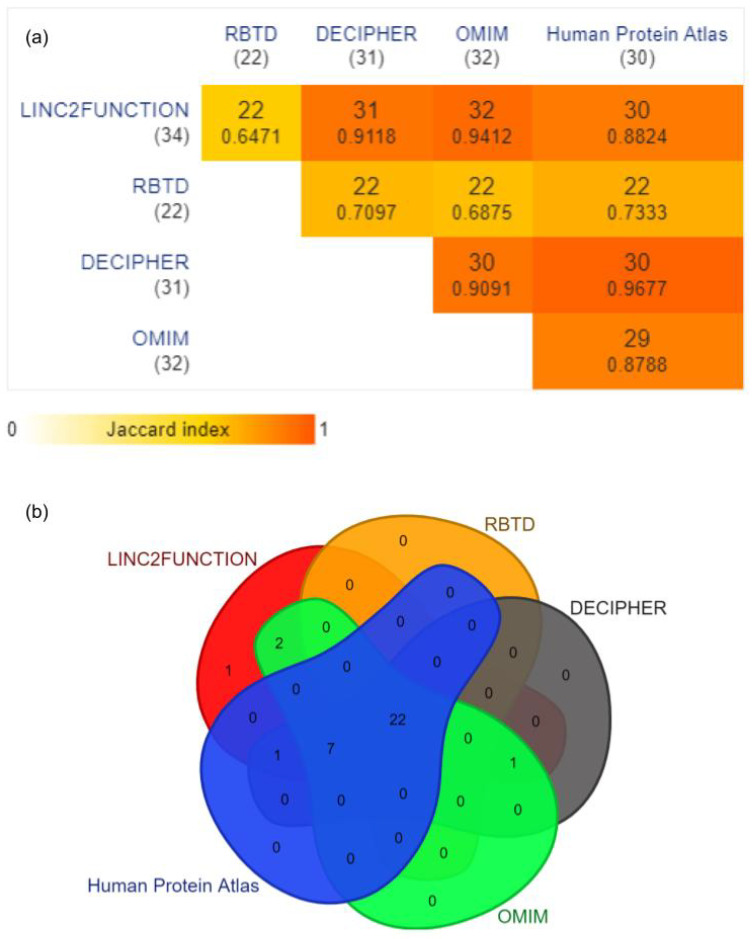
(**a**) The pairwise intersections of linc2function predicted RBPs with the above-mentioned databases. The data are represented as a minimal triangular matrix using the Jaccard index. (Generated using https://www.molbiotools.com/listcompare.php (accessed on 13 September 2023)). (**b**) Venn diagram showing the overlap for 34 unique RBP entries of linc2function with four different databases, namely RBTD (overlap 22/34 *p*-value: 1.9460 × 10^−4^ †), OMIM (overlap 32/34 *p*-value: 4.211210^−7^ †), Human Protein Atlas (overlap 30/34 *p*-value: 1.486510^−6^ †), and Decipher (overlap 31/34 *p*-value: 7.765510^−7^ †) (Generated using https://www.molbiotools.com/listcompare.php (accessed on 13 September 2023)). † The *p*-values were computed by conducting a hypergeometric test through the DynaVenn online tool [46].

**RNA-Binding Proteins (RBPs):** Thirty four unique RBPs predicted by *linc2function* were considered and verified through RBTD, DECIPHER, OMIM, and Human Protein Atlas. The results reported by *linc2function* show a 64.7% (22 out of 34) overlap with all four databases and a 88.2–94 % overlap with different databases individually, and only 2.9% RBPs (1 out of 34 Vts1) do not belong to any database (Figure 4).

RBPTD validation confirmed that *linc2function*-predicted RBPs play vital roles in tumour sites such as the uterus and the ovaries. While differential expression analysis was unavailable for the ovary site due to data limitations on the website, patient survival analysis data highlighted the significance of RBPs in those tissues. Among the RBPTD entries, 64.7% overlapped with the *linc2function*-predicted RBPs, and there were no exclusive entries. Among the predicted RBPs, 12 were not found in RBPTD, namely *RBMY1A1*, *sap-49*, *SFRS9*, *YTHDC1*, *SFRS13A*, *SFRS1*, *SFRS2*, *Vts1*, *A2BP1*, *Psi*, *SFRS7*, and *sus*.

Regarding the DECIPHER database, there was a 91.2% (31 out of 34) convergence between *linc2function*-predicted RBPs and the database. The DECIPHER database revealed the pathogenicity and phenotype of the query, and except for four RBPs, all others from *linc2function* demonstrated pathogenic effects with direct and indirect associations with prenatal developmental abnormalities and gynecological disorders such as retarded intrauterine growth and ovarian cysts. In summary, the pathogenicity and phenotypes of these genes indicate their crucial role in female reproductive organs, newborns, and reproductive mechanisms. The three excluded RBPs are *Vts1*, *Psi*, and *sus*.

*linc2function* exhibited a significant overlap of 94.1% (32 out of 34) with the OMIM database data. However, the information for *sap-49* and *Vts1* was missing in the cross-checked database. The considered RBPs were found to be associated with various carcinogenic mechanisms, such as tumour antigens, or were screened in different carcinoma cell expressions.

We also used the Human Protein Atlas, specifically the Pathology Atlas, which contains mRNA information for 17 forms of cancer. The comparison found an 88.2% (30 out of 34) overlap between the two lists. However, there were exceptions for the RBPs *RBMY1A1*, *Vts*, *Psi*, and *sus*. All other RBPs were identified as significant cancer prognostic markers or categorized as disease genes. Additionally, certain genes were listed as cancer genes, with three of them being specific to endometrial cancer (*NONO*, *SFRS2*, and *EIF4B*).

Furthermore, the pipeline’s prediction of RBP-binding sites reveals a high degree of conservation. To be exact, genomic locations identified as potential protein targets by *linc2function* exhibit an impressive conservation rate of 98.78% across six distinct species: human, chimp, rhesus, dog, mouse, rat, and opossum. These statistics validate and underscore the reliability of the *linc2function* pipeline, as depicted in Figure 4. The documentation offers a comprehensive, step-by-step guide for verifying sequence conservation at RBP binding sites. https://linc2function-tutorial.readthedocs.io/en/latest/conservation_pipeline.html (accessed on 13 September 2023).

**microRNA:** *linc2function* generates Target name, Target Length, Accessibility Energy, Hybridization Energy, Interaction Energy, and Base Pair for the predicted RNA interactome. The top 100 miRNA interactions for each lncRNA were selected based on the hybridization energy. The 486 unique miRNA interactions were listed and cross-checked with published CLIP-seq results. There was an overlap for 54 miRNAs, as they interact with 667 major unique target lncRNAs in various biological processes (a total of 2396 miRNA–lncRNA interactions), which underlines that its disease associations include the complete information on RNA interactome validation.

**Triplex forming potential:** One of the common ways that lncRNA and DNA interact is via direct triplex formation. According to linc2function prediction, four lncRNAs (ENSG00000228397.1, ENSG00000218510.3, ENSG00000230068.2, CATG00000083048.1) showed triplex-forming potential in the range of 0.0272–0.2195 and the rest of the lncRNAs showed 0 triplex-forming potential.

##### Summary

Thus, in this case study, we used the *linc2function* pipeline to annotate 17 lncRNAs associated with endometriosis. The analysis yielded confident RBPs and interaction regions. Validation involved mapping against four known disease databases. Our results exhibited a 64.7% overlap of linc2function-predicted RBPs with all databases, 88.2–94% with individual databases, and 2.9% potentially novel RBPs. MicroRNA interactions included 54 miRNAs’ overlapping target capture experiment (CLIP-seq) data. Four lncRNAs exhibited triplex formation potential, indicating their regulatory role involving RNA:DNA interactions. Overall, *linc2function*’s reliability was reinforced.

## 4. Conclusions

To the best of our knowledge, this is the first study to develop a pipeline for end-to-end lncRNA annotation comprising six different models. While the Table 1 lists tools for lncRNA identification, linc2function offers a comprehensive analysis, predicting functional characteristics through interactome and secondary structure analysis. Depending on the research question, one can select the model that works best for the analysis. The ANN approach allows pre-computed features, comprising sequences, structures, and interactomes, to be run as species-specific or species-agnostic models. This cross-species generalization ability of the species-agnostic models is unique to our approach, which makes it applicable to species that do not have a well-annotated reference genome yet. Additionally, the lightweight models allow a large number of nucleotide data to be screened quickly while also allowing for easy interpretation (refer to Supplementary Figures S1 and S2). On the other hand, the deep learning approach can predict lncRNA based only on the sequence, consistently achieving high AUC values exceeding 0.9 across eight different species.

Our models rely on well-curated high-confidence annotations from the latest genome build, enhancing their reliability. Notably, existing tools in this domain primarily offer prediction capabilities using pre-built models. Even when source code is available, the original training data are often inaccessible. In contrast, *linc2function* provides a modular and extensible framework, allowing users to retrain models with new features or on novel species. Furthermore, our open-access code promotes community reuse. This flexibility accommodates users with diverse computing infrastructure and can be accessed via a web interface or as a standalone installation, catering to a wide user base ranging from bioinformaticians to biologists.

Our findings underscore the shared biological characteristics of lncRNAs across species. Through our case studies, we have illustrated how *linc2function* can aid in the identification of disease-associated lncRNA isoforms. Specifically, we’ve showcased the potential for identifying lncRNA isoforms as disease biomarkers, emphasizing the importance of gaining a deeper understanding of their functional roles in disease etiology. *linc2function* also excelled in the complete annotation of lncRNA protein interactome, showing an overlap (RBPs) of about 94% with four databases. Further, the miRNA interactions obtained on the basis of hybridization energy consist of 54 miRNAs that overlap with the target capture experiment (CLIP-seq) data. Our work unveils, for the first time, the impact of alternative splicing on the functions of distinct lncRNA isoforms by altering their interactome-binding sites.

## Figures and Tables

**Table 1:** This table presents a summary of a short survey of various studies focused on predicting lncRNA, indicating the year of release, the machine learning model employed, and the types of features utilized. The types of features include Sequence-Based, Conservation, Structure, Experiment (e.g., expression), and Interactome. The method *linc2function* is highlighted in bold text for easy identification.

Tool	Year	ML Model	SB	Con	Str	Exp	Int
CPC	2007	SVM	y	y	n	n	n
CPAT	2013	LR	y	n	n	n	n
CNCI	2013	SVM	y	n	n	n	n
PLEK	2014	SVM	y	n	n	n	n
lncRScanSVM	2015	SVM	y	y	n	n	n
lncRNAID	2015	RF	y	y	n	n	n
lncRNAMFDL	2015	ANN	y	y	y	n	n
lncScore	2016	LR	y	y	n	n	n
DeepLNC	2016	ANN	y	n	n	n	n
CPC2	2017	SVM	y	n	y	n	n
COME	2017	RF	y	y	n	y	n
Longdist	2017	SVM	y	n	n	n	n
FEElnc	2017	RF	y	n	n	n	n
LncADeep	2018	DL	y	y	n	n	n
LncRNAnet	2018	CNN, RNN	y	n	n	n	n
EVlncRNApred	2019	SVM	y	y	y	y	y
LncFinder	2019	LR, SVM, RF, ELM, ANN	y	n	y	n	n
**linc2function**	**2023**	**ANN, CNN, RNN**	**y**	**n**	**y**	**n**	**y**

**Table 2:** Comparing the performance of the linc2function models and lncRNAnet. * A pre-trained model of lncRNAnet was used. It was likely that the sequences used in the prediction were part of training data too. Hence, this can explain the slightly inflated performance metrics as compared to the original publication.

Model	Precision	Recall	Specificity	F1	AUROC
Linc2function HSS	0.8771	0.8852	0.8760	0.8812	0.949
Linc2function SAS	0.8717	0.8625	0.8730	0.8671	0.941
Linc2function HSB	0.8592	0.8815	0.8555	0.8702	0.936
Linc2function SAB	0.8591	0.8702	0.8572	0.8646	0.933
Linc2function LRN2	0.9412	0.9160	0.9428	0.9284	0.967
LncRNAnet *	0.9750	0.8962	0.9770	0.9340	0.977

## Data Availability

The datasets under analysis are openly accessible https://bioinformaticslab.erc.monash.edu/datasource_comparison(accessed on 13 September 2023). You can find the source code at https://gitlab.com/tyagilab/linc2functionpipeline (accessed on 13 September 2023), access the pipeline at https://bioinformaticslab.erc.monash.edu/linc2function (accessed on 13 September 2023), and refer to the documentation provided at https://linc2function-tutorial.readthedocs.io/en/latest/ (accessed on 13 September 2023).

## Supplementary Materials

The following supporting information can be downloaded at https://www.mdpi.com/article/10.3390/epigenomes7030022/s1. Figure S1: SHAP plot showing feature importance values for HSB model. Figure S2: SHAP plot showing feature importance values for SAB model. Figure S3: Recursive Feature Elimination performed for selecting the optimum feature set for building machine learning models. Figure S4: Venn diagram showing the percentage overlap of LncRNAs between LncBook, LNCipedia, GENCODE, and NONCODE obtained by performaing genomic approxiamate co-ordinates comparison. Figure S5: Figure illustrating the sliding window approach where secondary structure is predicted for smaller overlapping sequences are stitched together to obtain the structure of a larger sequence. Figure S6: Figure showing the web interface of linc2function utility. Figure S7: The cross-species analysis, prediction performance, measured by the AUROC values, of linc2function models is assessed across eight different species: Fruit Fly, Human, Mouse, Roundworm, Sea Vase, Wheat, Yeast, and Zebrafish. Furthermore, each bar is labeled with its corresponding AUROC and the 95% confidence interval. Additionally, the standard errors are depicted as whiskers atop the bars. Tables S1–S8 see the supplementary file.
