# Supplementary material for: Linc2function: A Comprehensive Pipeline and Webserver for Long Non-Coding RNA (lncRNA) Identification and Functional Predictions Using Deep Learning Approaches
Source: Epigenomes. 2023 Sep 15;7(3):22. doi: 10.3390/epigenomes7030022 (PMC10528440; doi:10.3390/epigenomes7030022)
Supplement: Supplementary file 1 [file epigenomes-07-00022-s001.zip › linc2function_supplementary.pdf]

Supplementary Material

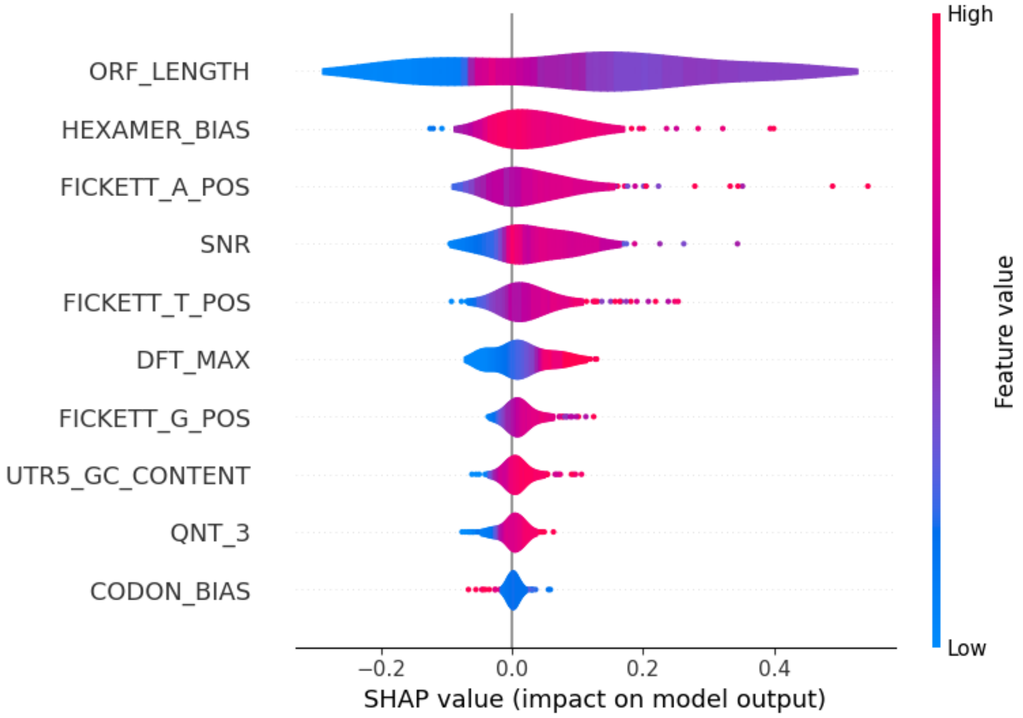

Figure S1. SHAP plot showing feature importance values for HSB model

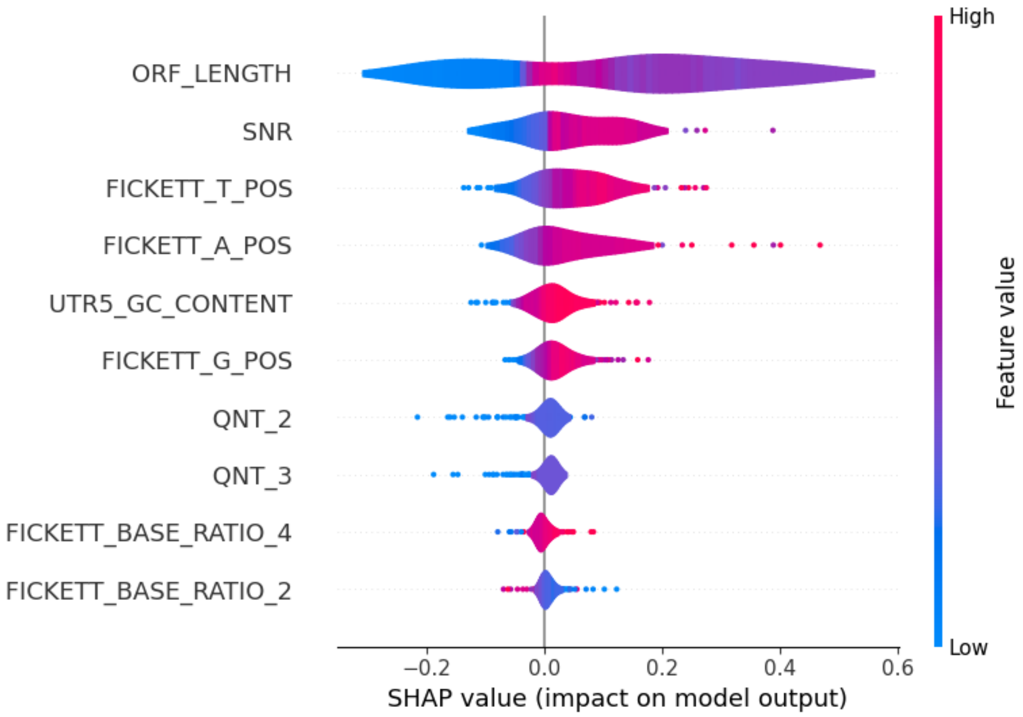

Figure S2. SHAP plot showing feature importance values for SAB model

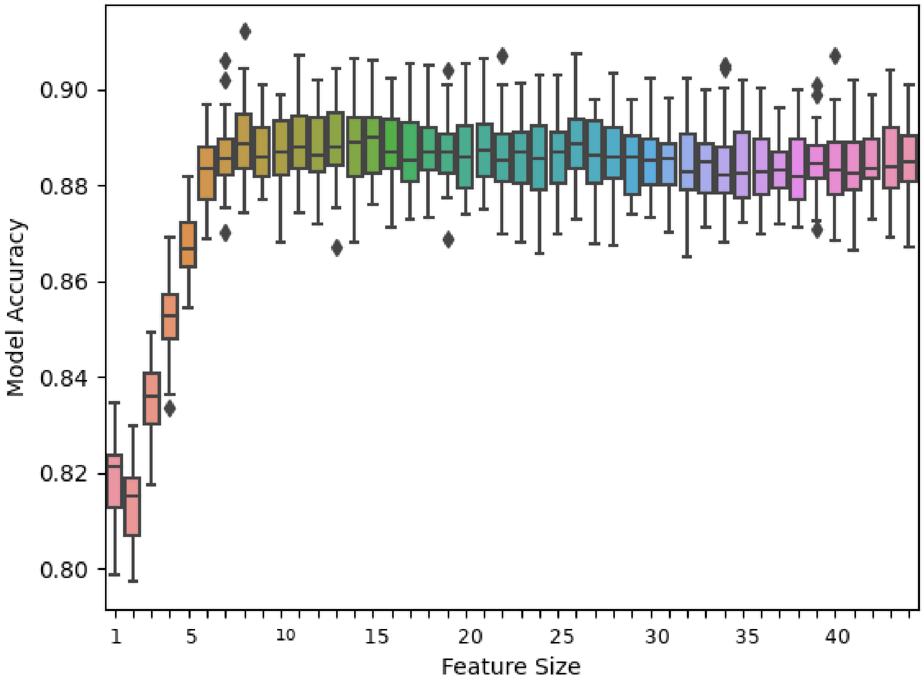

Figure S3. Recursive Feature Elimination performed for selecting the optimum feature set for building machine learning models

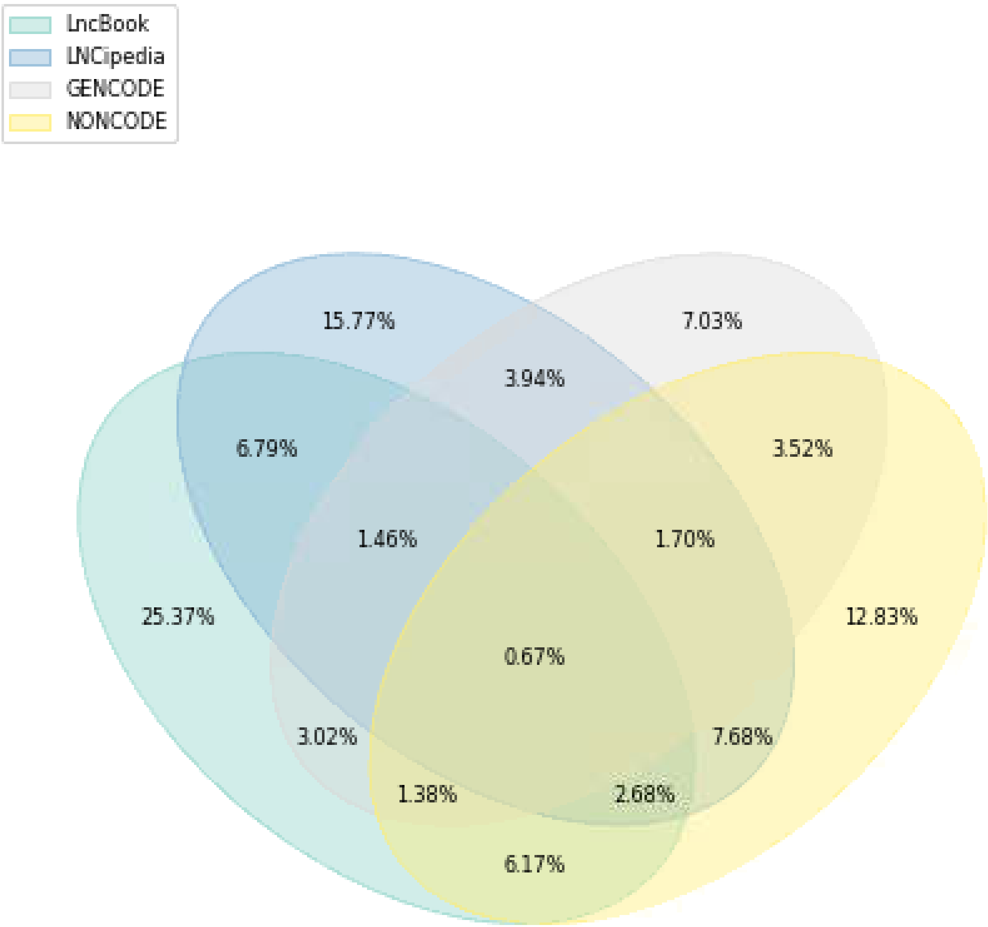

Figure S4. Venn diagram showing the percentage overlap of LncRNAs between LncBook, LNCipedia, GENCODE, and NONCODE obtained by performaing genomic approxiamate co-ordinates comparison

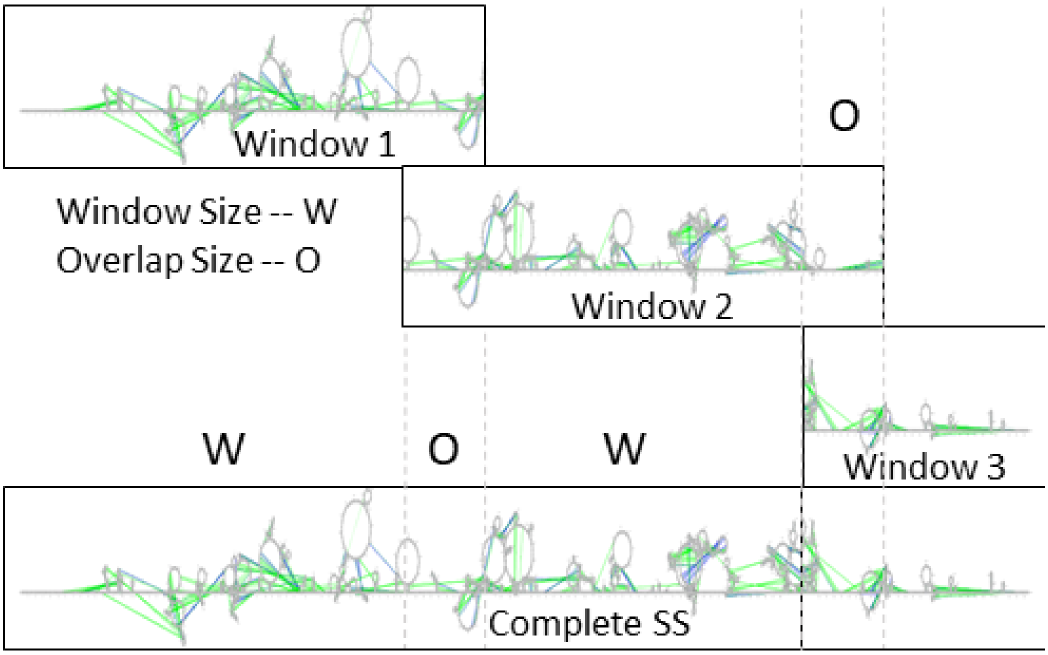

Figure S5. Figure illustrating the sliding window approach where secondary structure is predicted for smaller overlapping sequences are stitched together to obtain the structure of a larger sequence.

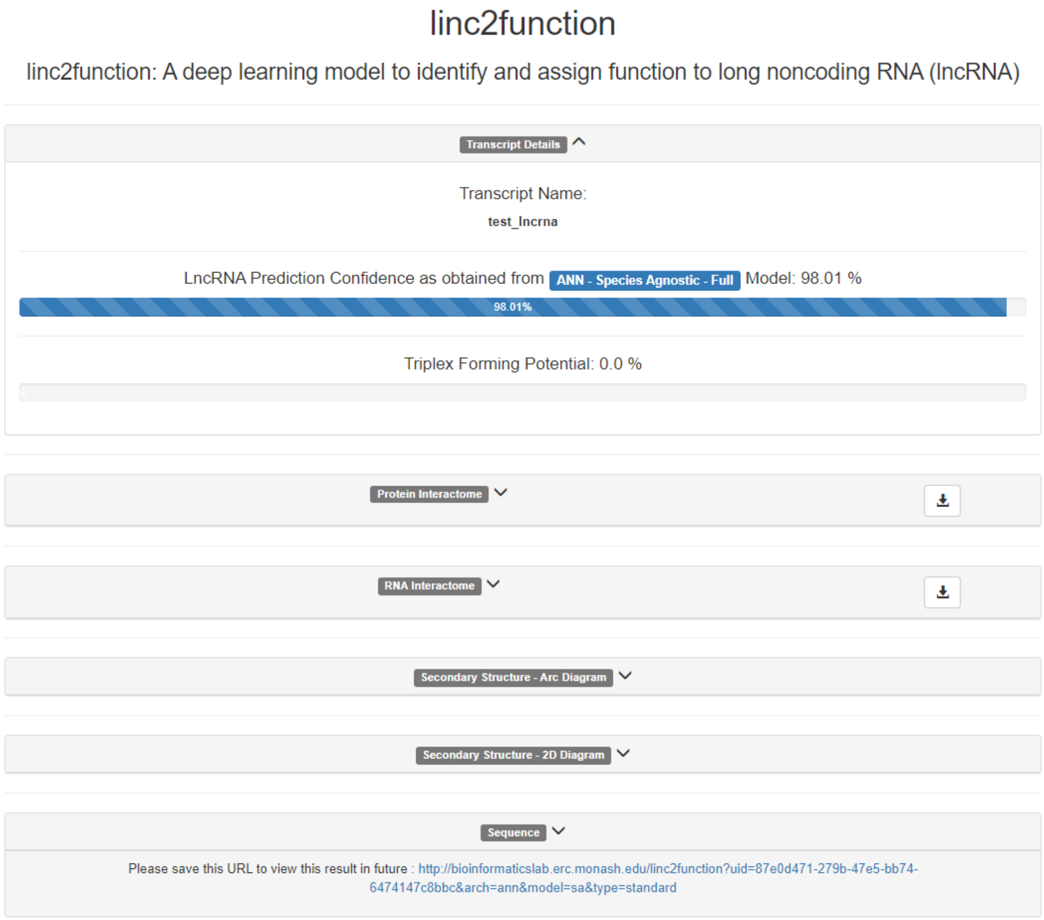

Figure S6. Figure showing the web interface of linc2function utility.

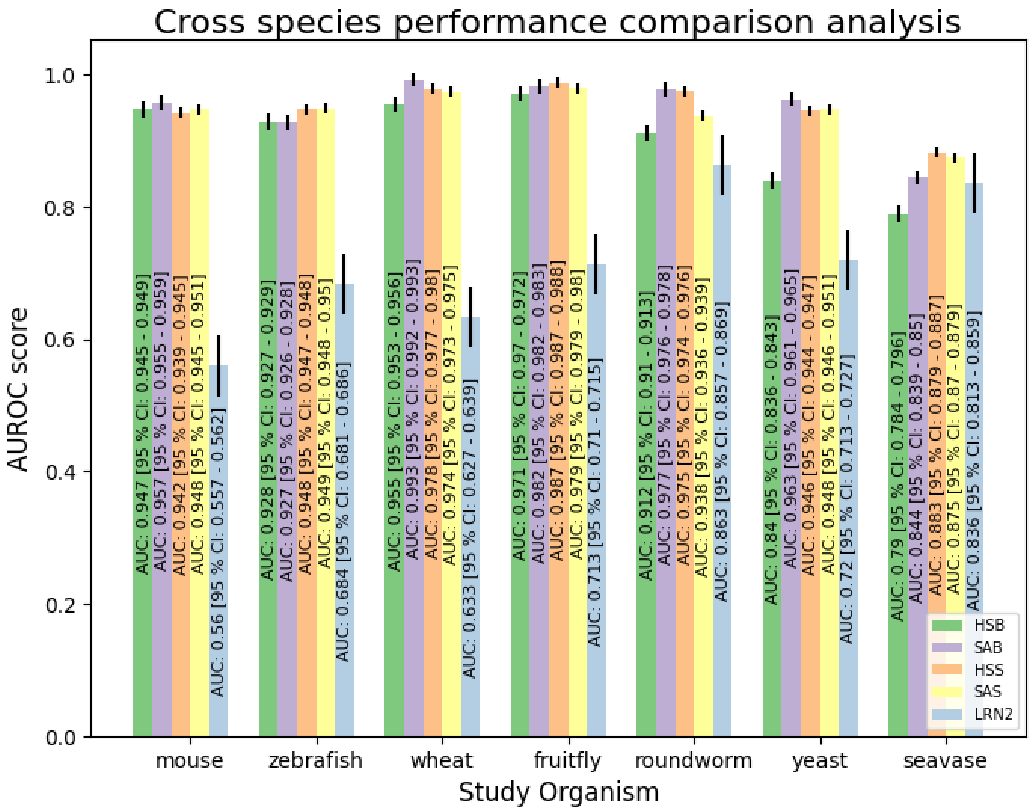

Figure S7. The cross-species analysis, prediction performance, measured by the AUROC values, of linc2function models is assessed across eight different species: Fruit Fly, Human, Mouse, Roundworm, Sea Vase, Wheat, Yeast, and Zebrafish. Furthermore, each bar is labeled with its corresponding AUROC and the 95% confidence interval. Additionally, the standard errors are depicted as whiskers atop the bars.
